# Unexpected occult malignancy diagnosed during tonsillectomy surgery for obstructive sleep apnea: A case report

**DOI:** 10.1097/MD.0000000000019793

**Published:** 2020-05-01

**Authors:** Donghwi Park, Byung Joo Lee, Minchul Go, Jung-Soo Kim, Sung Jae Heo

**Affiliations:** a Department of Physical Medicine and Rehabilitation, Ulsan University Hospital, University of Ulsan College of Medicine, Ulsan; b Department of Otorhinolaryngology – Head and Neck Surgery, School of Medicine, Kyungpook National University, Kyungpook National University Hospital; c Department of Otorhinolaryngology – Head and Neck Surgery, School of Medicine, Kyungpook National University, Kyungpook National University Chilgok Hospital, Daegu, South Korea.

**Keywords:** apnea, human papillomavirus 16, oropharyngeal neoplasms, squamous cell carcinoma of the head and neck, tonsillectomy

## Abstract

**Rationale:**

Many previous studies have investigated the necessity of routine histopathological analysis of tonsillectomy specimen, and most recent studies have suggested that such an analysis is not justified in asymptomatic patients or those with no risk factors for malignancy.

**Patient concerns:**

A 59-year-old man diagnosed with obstructive sleep apnea underwent surgery, including tonsillectomy; a tonsil specimen was sent to the department of pathology.

**Diagnosis:**

Although the patient did not exhibit any tonsil-related signs or symptoms, nor did the appearance of the tonsil appear to be pathological, the right tonsil specimen was diagnosed with squamous cell carcinoma, with detection of human papilloma virus 16.

**Interventions:**

Chemotherapy and radiotherapy were used to treat the tonsil cancer.

**Outcomes:**

No recurrence was observed during the 4-year follow-up.

**Lessons:**

In the future, it may be necessary to reinvestigate the necessity of routine histopathological analysis of tonsillectomy specimens in asymptomatic patients, considering the drastically increased rate of detection of human papilloma virus-related oropharyngeal squamous cell carcinomas in these patients.

## 1 Introduction

Obstructive sleep apnea (OSA) is one of the most common indications for tonsillectomy in the adult population.^[1]^ Despite the benign indications for surgery in the vast majority of cases, such as OSA, tonsillectomy specimens are routinely sent for gross and histopathological evaluation for detection of occult malignancy.^[2]^

In the adult patient population, the incidence of malignancy in tonsillectomy specimens has been reported to range from 2% to 10%.^[2,3]^ In these previous studies; however, none of the malignancies were unexpected, except for 1 case (unexpected malignancy in tonsillectomy specimen from a 46-year-old man^[2]^). Furthermore, most of these patients had at least 1 risk factor, including history of head and neck cancer; marked tonsil asymmetry; pathological firmness; enlargement of neck lymph node; unexplained weight loss and constitutional symptoms; or visible lesion on the tonsil (ie, ulcer).^[2,4]^ Many previous studies have investigated the necessity of routine histopathological analysis of tonsillectomy specimen, and most recent studies have suggested that such an analysis in asymptomatic patients and/or those with no risk factors for malignancy is not justified because the probability of identifying an unexpected occult malignancy is exceedingly low.^[2,4]^ Nevertheless, we report an unexpected occult malignancy in a tonsillectomy specimen from a patient with OSA who had no risk factors for malignancy. This report may also prompt reconsideration of the necessity of routine histopathological analysis of tonsillectomy specimen in patients without risk factors for malignancy.

This case report was approved by ethics committee of the Kyungpook National University Hospital and informed consent was obtained from the patient for publication of this report.

## 2 Case report

A 59-year-old man visited the authors’ department complaining of sleep apnea, snoring, and frequent wakefulness during sleep. He did not have any underlying disease. On endoscopic examination, tonsil size and tongue position were grade I and III, respectively, according to the Friedman staging system. He was diagnosed with OSA based on the results of type I polysomnography (respiratory distress index, 21.4; no central respiratory events observed). Treatment with positive airway pressure was initially prescribed; however, the patient failed to adjust to the therapy and preferred, instead, to undergo surgery. Therefore, anterior pharyngoplasty with tonsillectomy, partial uvulectomy, and tongue base reduction were performed.

**Figure 1:**
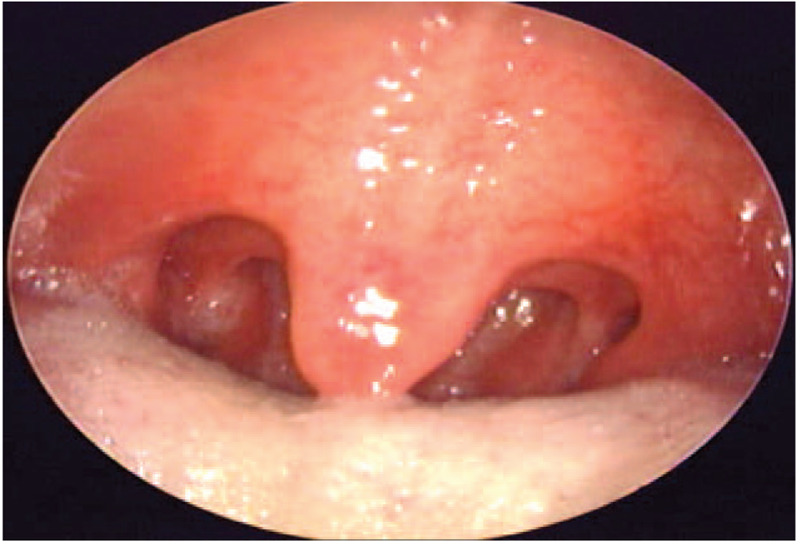
Preoperative endoscopic findings of palatine tonsil.

**Figure 2:**
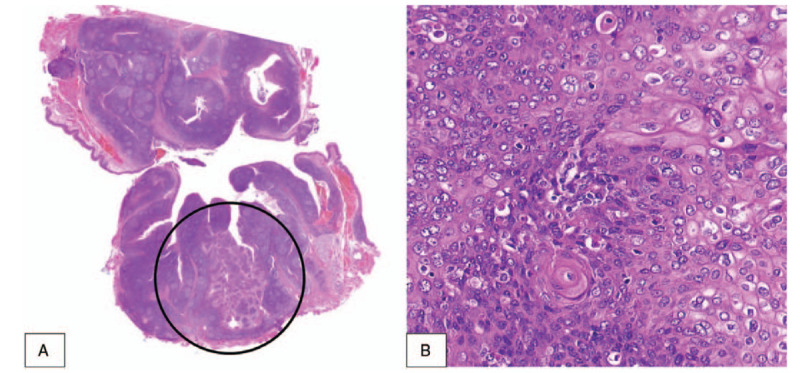
Histopathological findings. Squamous cell carcinoma (black circle) in the lymphoid tissue of tonsil is shown in A (×2). Atypical squamous cells with severe variation of size and nuclear pleomorphism are shown in B, which is a finding consistent with squamous cell carcinoma (×200).

A tonsil specimen was sent to the department of pathology, and the patient was discharged without complications on postoperative day 3. Although he did not exhibit any tonsil-related signs or symptoms, and the tonsil did not have a pathologic appearance (Fig. 1), a biopsy specimen from the right tonsil was diagnosed with squamous cell carcinoma (SCC) (Fig. 2). The resection margin was clear, and genotyping for 28 types of human papilloma virus (HPV) detected HPV 16 in the tonsil specimen. Metastasis of the tumor was not observed on computed tomography and positron emission tomography. Chemotherapy (cisplatin 166.3 mg and 161.4 mg for 1st and 2nd cycle, respectively) and radiotherapy (66 Gy) were used to treat the tonsil cancer. Surgical sites in the oral cavity were completely cancer-free after treatment, and no recurrence of tonsil cancer was observed during the 4-year follow-up. OSA was cured (respiratory distress index, 3.3) and symptoms (snoring and frequent wakefulness during sleep) improved after sleep surgery.

## 3 Discussion

To date, more than 200 HPV genotypes have been identified.^[5]^ Mucosal HPV types have been categorized into “high-risk HPV” (HR-HPV) and “low-risk HPV” types according to their potential to induce malignancy in the cervix.^[5]^ Twelve HR-HPV types have been classified as oncogenic by the International Agency for Research on Cancer (HPV genotypes 16, 18, 31, 33, 35, 39, 45, 51, 52, 56, 58, and 59).^[5]^ Among the HR-HPV types involved in head and neck cancer, HPV16 is, by far, the most common, with a prevalence > 80% in patients diagnosed with oropharyngeal SCC (OPSCC), followed by HPV18 (3%).^[6]^ In addition, the International Agency for Research on Cancer has estimated that approximately 31% of OPSCC cases are due to HPV.^[7]^ In our case, the patient also was infected with HPV16, although there were no other symptoms aside from OSA. Considering that HPV16 is a high-risk carcinogen and the most common HR-HPV type in OPSCC, the patient was diagnosed with incidental SCC due to HPV16 infection.

As mentioned, many previous studies have exhorted the diminishing role of histopathological analysis of tonsillectomy specimens in benign tonsillectomy surgery, with evidence supporting only gross analysis. In the adult patient population, the prevalence of unsuspected tonsillar malignancy in routine tonsillectomy specimens has been reported to be very low (0.03%; 1 case of squamous cell carcinoma out of 3904 cases,^[2]^ and 0.015%; 9 cases of lymphoma and 2 cases of squamous cell carcinoma out of 72,322 cases^[4]^). However, given that most cases of malignant tonsil cancer are found after metastasis, it is also true that there is concern about not performing routine histopathological analysis of tonsillectomy specimens due solely to cost-effectiveness in real-world clinical situations. Moreover, it is also possible that whereas the current yield in using benign tonsil specimens to screen for carcinoma is exceptionally low, over time, it may increase due to the rising incidence of HPV-related oropharyngeal carcinomas. In fact, the prevalence of HPV-related OPSCC has increased drastically in the past decade in some developed countries.^[5]^ Considering this increased prevalence, it may be necessary to reinvestigate this issue by addressing the incidence of OPSCC and the disadvantages of missing a cancer, and compare these with the cost of cancer care in the future.

In summary, in the future, it may be necessary to reinvestigate the necessity of routine histopathological analysis of tonsillectomy specimens in asymptomatic patients, considering the recent drastic increase in the frequency of detection of HPV-related OPSCCs in these patients.

## Author contributions

**Conceptualization:** Byung Joo Lee, Sung Jae Heo.

**Data curation:** Minchul Go, Jung-Soo Kim, Sung Jae Heo.

**Formal analysis:** Donghwi Park, Byung Joo Lee, Minchul Go, Jung-Soo Kim, Sung Jae Heo.

**Writing – original draft:** Donghwi Park, Byung Joo Lee.

**Writing – review and editing:** Donghwi Park, Byung Joo Lee, Sung Jae Heo.
